# Construction and validation of a fatty acid metabolism risk signature for predicting prognosis in acute myeloid leukemia

**DOI:** 10.1186/s12863-022-01099-x

**Published:** 2022-12-22

**Authors:** Miao Chen, Yuan Tao, Pengjie Yue, Feng Guo, Xiaojing Yan

**Affiliations:** 1 Department of Hematology, The First Affiliated Hospital of China Medical University, Liaoning 110001 Shenyang, China; 2 Department of Pharmaceutical Toxicology, School of Pharmacy, China Medical University, Shenyang, Liaoning 110122 China

**Keywords:** Acute myeloid leukemia, Fatty acid metabolism, Prognostic signature, Mitochondrial metabolism

## Abstract

**Background:**

Fatty acid metabolism has been reported to play important roles in the development of acute myeloid leukemia (AML), but there are no prognostic signatures composed of fatty acid metabolism-related genes. As the current prognostic evaluation system has limitations due to the heterogeneity of AML patients, it is necessary to develop a new signature based on fatty acid metabolism to better guide prognosis prediction and treatment selection.

**Methods:**

We analyzed the RNA sequencing and clinical data of The Cancer Genome Atlas (TCGA) and Vizome cohorts. The analyses were performed with GraphPad 7, the R language and SPSS.

**Results:**

We selected nine significant genes in the fatty acid metabolism gene set through univariate Cox analysis and the log-rank test. Then, a fatty acid metabolism signature was established based on these genes. We found that the signature was as an independent unfavourable prognostic factor and increased the precision of prediction when combined with classic factors in a nomogram. Gene Ontology (GO) and gene set enrichment analysis (GSEA) showed that the risk signature was closely associated with mitochondrial metabolism and that the high-risk group had an enhanced immune response.

**Conclusion:**

The fatty acid metabolism signature is a new independent factor for predicting the clinical outcomes of AML patients.

**Supplementary Information:**

The online version contains supplementary material available at 10.1186/s12863-022-01099-x.

## Background

Acute myeloid leukemia (AML) is a hematopoietic neoplasm characterized by the clonal expansion of abnormally differentiated myeloid progenitor cells [1, 2]. With standard chemotherapy, AML patients have poor outcomes and high mortality rates because of relapsed disease and leukemia-related complications, especially in patients aged 60 years and older. In addition, the outcome of AML is heterogeneous with patient-related and disease-related factors [2, 3]. Currently, cytogenetic risk combined with molecular abnormalities is used as a classic risk stratification system to predict the probability of complete response (CR) and relapse, as well as overall survival (OS) according to the national recommendations [4, 5]. However, this system has limitations in patients without defined chromosomal or genetic alterations. Therefore, the development of a more accurate risk stratification system for AML is imperative to select suitable therapies and precisely predict clinical outcomes.

Metabolic reprogramming is a dynamic process accompanied by the whole process of leukemia [6–8]. When glucose metabolism shifts to aerobic glycolysis, AML cells enter a malignant proliferation phase, and when glucose metabolism shifts back into mitochondrial metabolism, AML cells enter a stem cell-based self-maintenance phase [9, 10]. Moreover, fatty acid metabolism also plays an important role in AML progression [11]. Specific alterations in fatty acid oxidation (FAO) and fatty acid synthesis (FAS) participate in core mitochondrial metabolic pathways influencing the fate of leukemia stem cells (LSCs), the adaptation to a specialized microenvironment, and the response to drugs. The expression of FAO enzymes including APOC2, CD36, CT2, FABP4, PHD3 and CPT1 were elevated in AML compared to normal hematopoiesis, moreover inhibition of these enzymes resulted in increased sensitivity to chemotherapy and decreased AML survival [12–17]. However, no modelled signature of fatty acid metabolism has been developed to predict the prognosis of AML patients and to further select therapeutic strategies based on fatty acid metabolism.

In this study, we established a fatty acid metabolism risk signature with significant prognostic value based on The Cancer Genome Atlas (TCGA) AML database and validated it in another AML database (Vizome). The fatty acid metabolism risk signature could independently identify AML patients with poor clinical outcomes more precisely than other prognostic markers.

## Results

### Construction of a fatty acid metabolism signature in AML

**Fig. 1:**
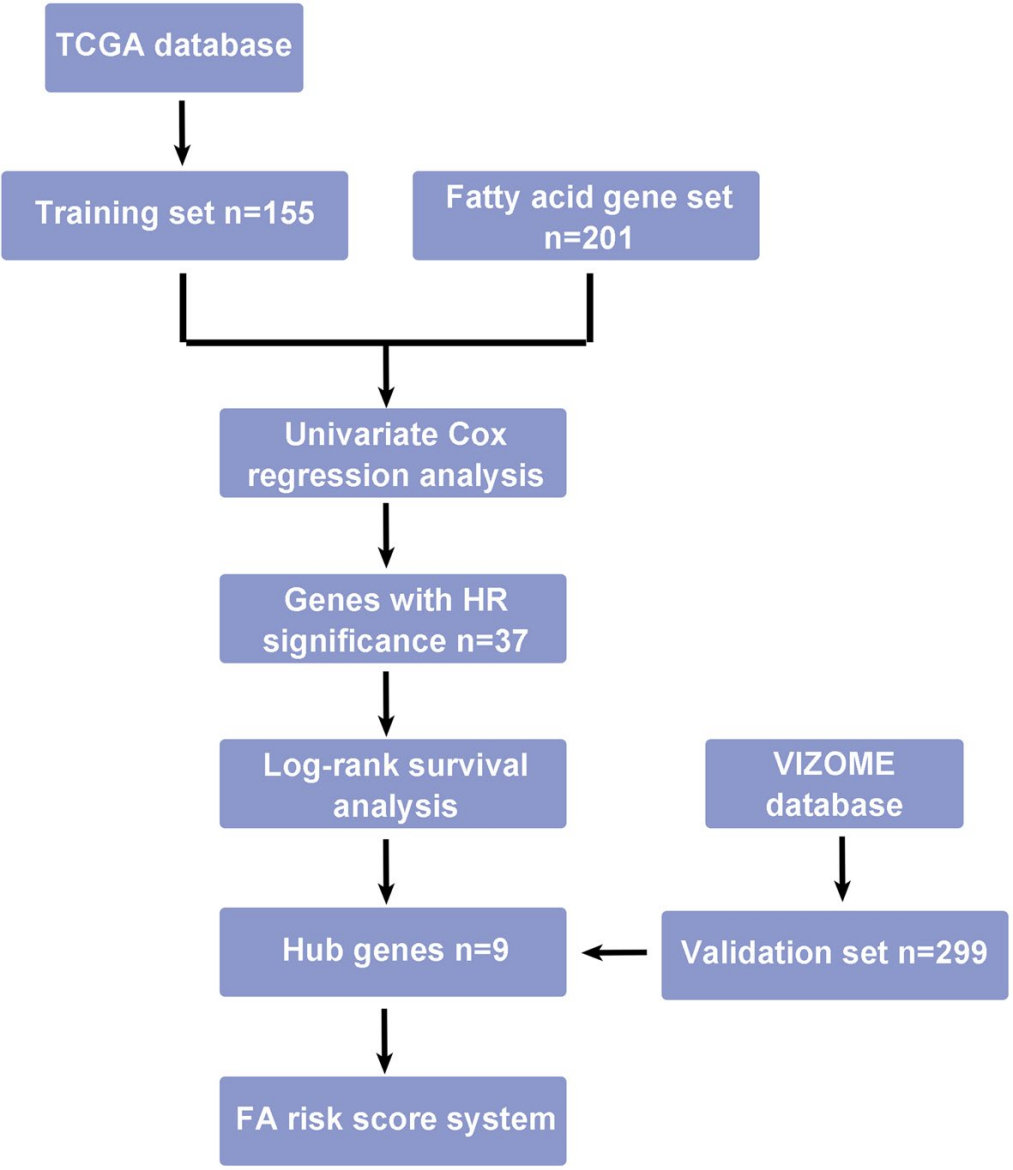
The flowchart of the signature construction

Considering the essential role of fatty acid metabolism in AML, we sought to establish a fatty acid metabolism signature (FA risk score) for prognostication. We used patients from the TCGA AML database as the training cohort. Univariate Cox regression analysis was used to explore the prognostic value of fatty acid metabolism-related genes (Supplementary Table 1). Thirty-seven genes were found to be associated with prognosis in AML (Supplementary Table 2 ). Then, we further screened the significant genes by log-rank prognostic analysis (Supplementary Fig. 1A) and finally selected 9 genes (MLYCD, CYP4F2, SLC25A1, PLA2G4A, ACBD4, ACOT7, ACSF2, CBR1, and ACSL5). MLYCD and CYP4F2 were identified as protective factors with hazard ratios (HRs) < 1, whereas SLC25A1, PLA2G4A, ACBD4, ACOT7, ACSF2, CBR1 and ACSL5 were defined as risk factors with HRs > 1 (Table 1). The procedure is illustrated in Fig. 1.

We then used the risk score method to establish a risk signature for patients with AML based on the gene expression levels as follows: FA risk score = (0.299 * SLC25A1 expression) - (1.090 * MLYCD expression) - (0.394 * CYP4F2A expression) + (0.474 * PLA2G4A expression) + (0.488 * ACBD4 expression) + (0.538 * ACOT7 expression) + (0.566 * ACSF2 expression) + (0.632 * CBR1 expression) + (0.750 * ACSL5 expression). The patients were divided into high-risk and low-risk groups based on the median risk score as the cut-off (Supplementary Fig. 1B).

**Table 1:** Cox Regression Analysis of TCGA RNA Sequencing Database, AML

Gene	HR	Low 95%	High 95%	*P* value
MLYCD	0.336	0.198	0.570	< 0.0001
CYP4F2	0.674	0.531	0.856	0.0012
SLC25A1	1.349	1.058	1.721	0.0159
PLA2G4A	1.606	1.291	1.997	< 0.0001
ACBD4	1.629	1.017	2.610	0.0425
ACOT7	1.712	1.249	2.346	0.0008
ACSF2	1.761	1.201	2.583	0.0038
CBR1	1.881	1.451	2.439	< 0.0001
ACSL5	2.116	1.320	3.392	0.0018

### Identification of the fatty acid metabolism signature as a prognostic marker in AML

**Fig. 2:**
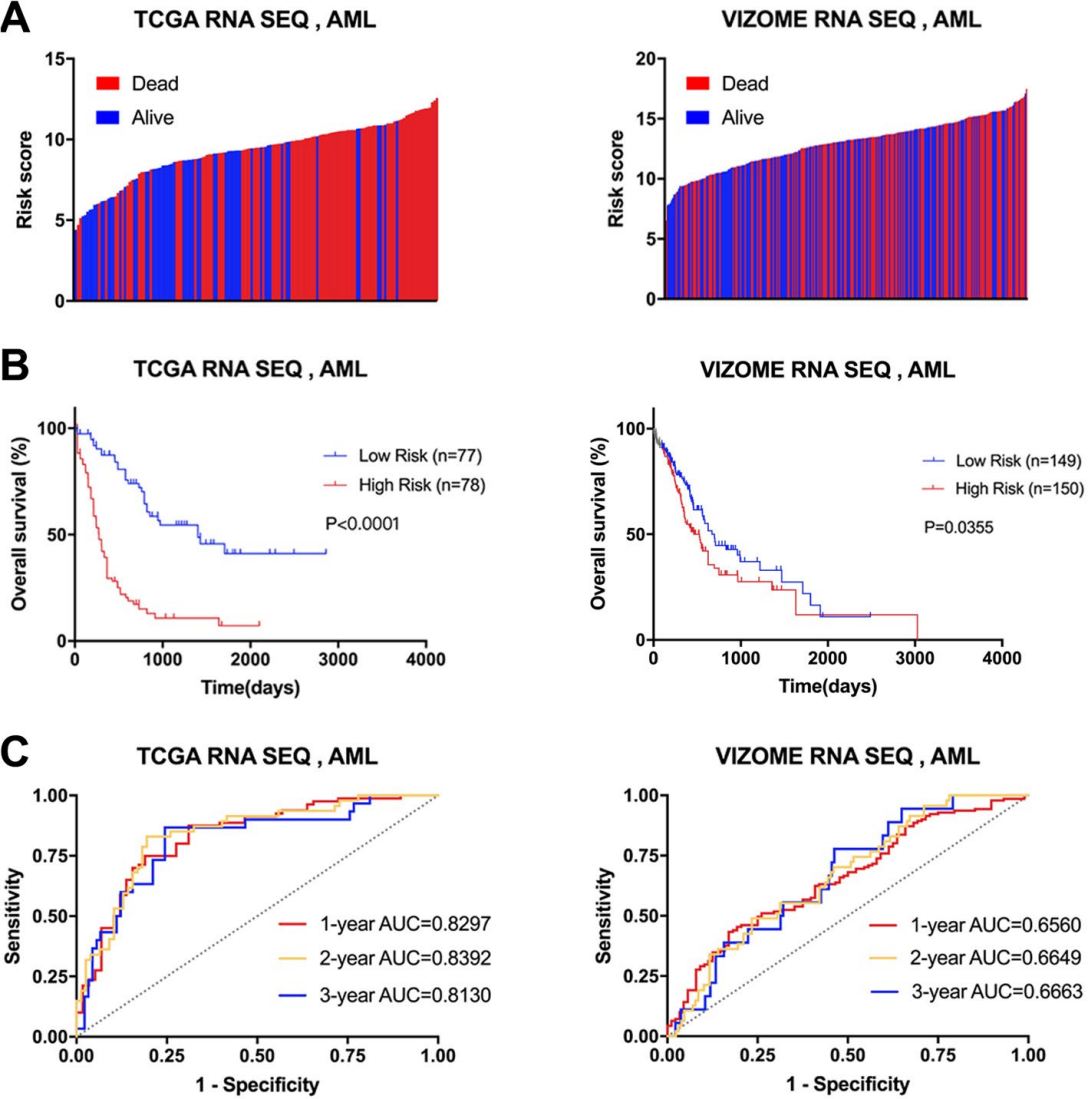
Prognostic value of the fatty acid metabolism signature in AML. **A** Survival outcome analysis of FA score distribution in training and validation cohort. **B** Kaplan-Meier analysis revealed the signature expressed prognostic value of AML in training and validation cohort (with log-rank test). **C** The time-dependent ROC curves showed the sensitivity and specificity of predicting 1-, 2- and 3-year overall survival according to the signature in training and validation cohort

We first analyzed the distribution of FA risk scores in patients with different survival statuses using a waterfall plot. Patients with lower FA risk scores generally had better survival outcomes (alive) than those with high risk scores (Fig. 2A). Then, we found that high-risk patients had shorter OS times than low-risk patients by log-rank analysis (Fig. 2B). To demonstrate the validity of the 9-gene FA metabolism risk signature in other independent populations, we calculated the risk score for each patient in the Vizome AML database [18] as an external cohort with the same formula. The patients were classified into high-risk and low-risk groups based on the median risk score. Consistent with the findings from the TCGA cohort, more surviving patients appeared in the low-risk group, and the OS time was shorter for high-risk patients than for low-risk patients (Fig. 2A-B). Moreover, the sensitivity and specificity of the FA risk score were assessed through time-dependent receiver operating characteristic (ROC) analysis. The areas under the curve (AUCs) for 1-, 2-, and 3-year OS were 0.8297, 0.8392 and 0.8130, respectively, in the training cohort, with significant *p* values (Fig. 2C). For validation in the external cohort, the AUCs for 1-, 2-, and 3-year OS were 0.6560, 0.6649 and 0.6663, respectively (Fig. 2C).

To explore the prognostic value of the fatty acid metabolism signature in stratified cohorts, the patients were classified by two traditional independent markers, age and cytogenetic risk. In the training cohort, high-risk patients had shorter OS times than low-risk patients in all stratified cohorts (Supplementary Fig. 2A-B). However, when we confirmed the results in the validation cohort, we found that the FA score only further predicted the prognosis in patients aged ≤ 60 years or with intermediate cytogenetic risk (Supplementary Fig. 2C-D). Overall, these results indicated that the FA signature is a prognostic marker in AML.

### The fatty acid metabolism signature is an independent risk factor for precisely predicting the survival time of AML patients

**Fig. 3:**
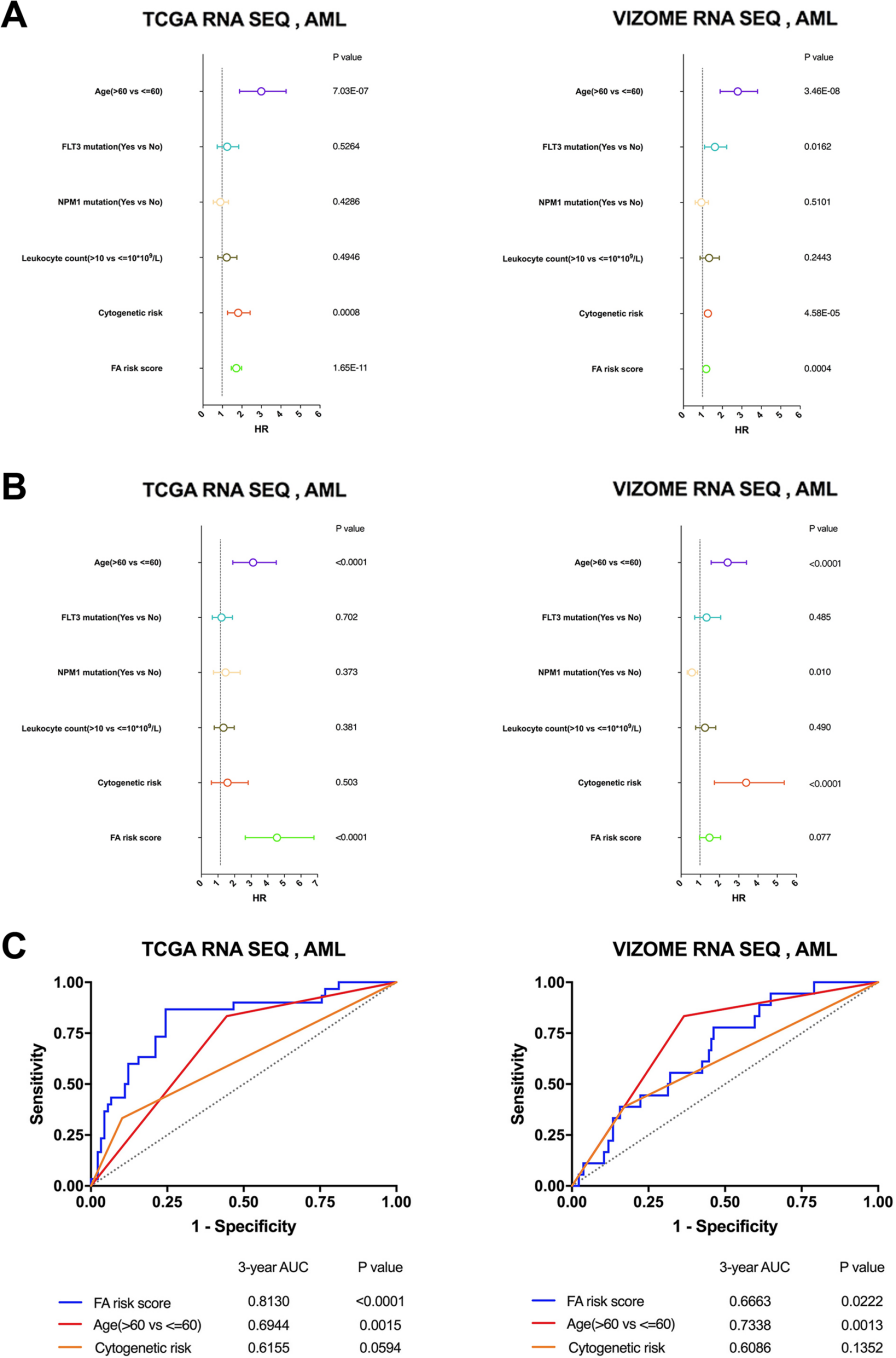
Comparing the fatty acid metabolism signature with classic prognostic factors. **A** Forest plots of univariate cox regression analysis in training and validation cohort. **B** Forest plots of multivariate cox regression analysis in training and validation cohort. **C** The time-dependent ROC curves showed the sensitivity and specificity of predicting 3-year overall survival according to the signature, age or cytogenetic risk in training and validation cohort

We next performed univariate and multivariate Cox regression analyses to determine whether the FA risk score is independently correlated with the OS of AML patients. We analyzed the prognostic value of the FA risk score together with other common prognostic factors (age, FLT3 mutation, NPM1 mutation, leukocyte count and cytogenetic risk). We found that the FA risk score served as an independent prognostic factor with an HR of 4.238 (*p* < 0.0001) in the training cohort and 1.406 (*p* = 0.077) in the validation cohort (Fig. 3A-B). Then, we conducted ROC curve analyses of the FA risk score and two other independent factors (age and cytogenetic risk) for predicting 3 years of OS in the training and validation cohorts and found that the AUC of the FA risk score was larger than that of cytogenetic risk or age (Fig. 3C). These findings confirmed the power of the FA risk score to independently predict prognosis in AML.

**Fig. 4:**
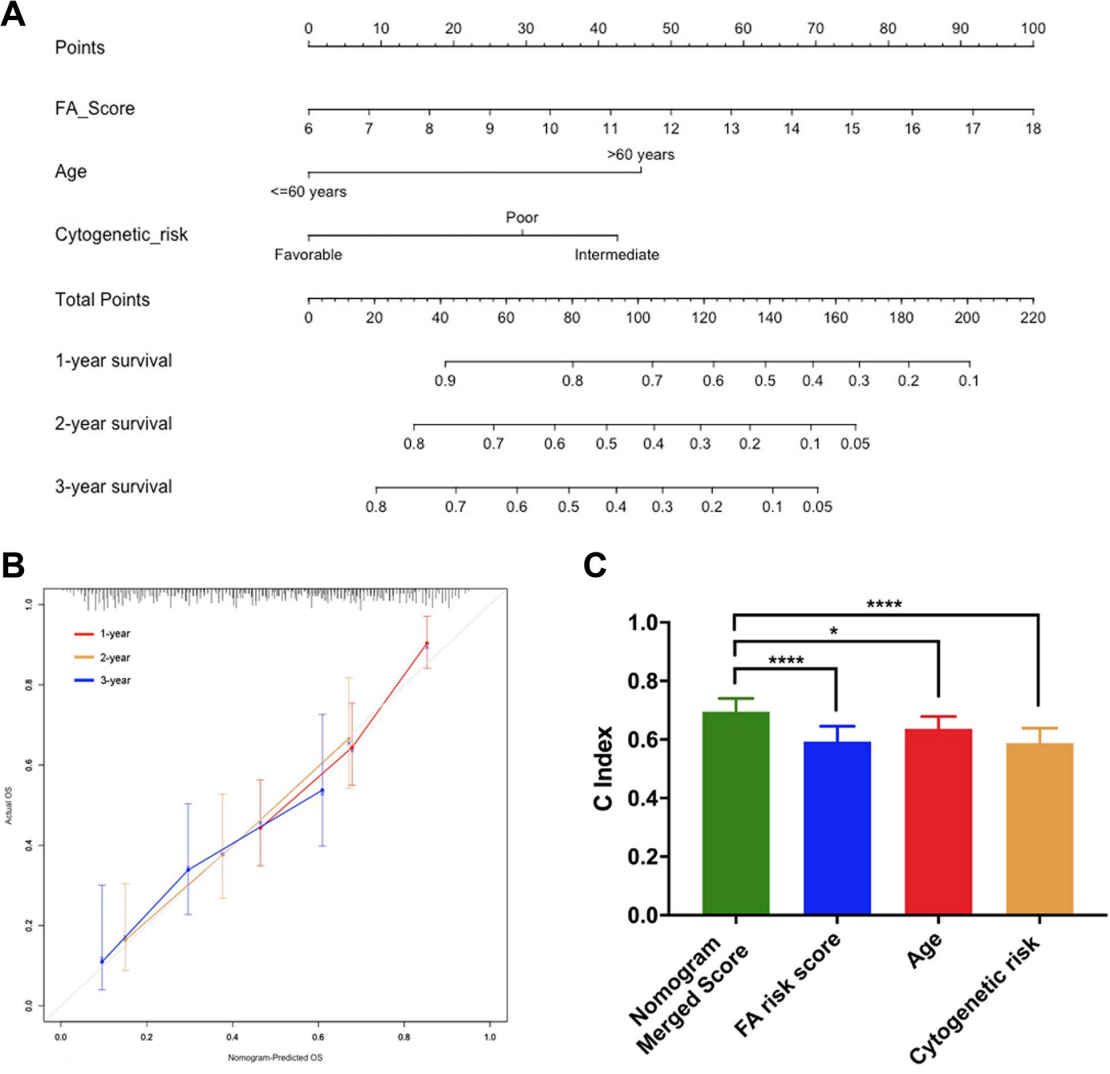
The nomogram combined the fatty acid metabolism signature and classic prognostic factors to predict the overall survival. **A** Nomogram plot showed the merged score system composed of the signature, age and cytogenetic risk in validation cohort. **B** Calibration plot showed the consistency of nomogram-predicted OS and actual OS in validation cohort. **C** The C-index comparison between the merged score and its single composition in validation cohort (with t test). *, *P* < 0.05; ****, *P* < 0.0001

To achieve a better translational and predictive evaluation system, we developed a nomogram integrating age, cytogenetic risk and FA score in the training set and validation set (Fig. 4A and Supplementary Fig. 3A). The calibration plots showed high concordance between the predicted and actual probabilities of 1-, 2- and 3-year survival (Fig. 4B and Supplementary Fig. 3B). The C-index of the merged nomogram score in the validation set was 0.7, which was significantly higher than that of its constituting factors (Fig. 4C). However, in the training set, the C-index of the merged nomogram score was close to the C-index of the FA score but higher than that of age and cytogenetic risk (Supplementary Fig. 3C). These results suggested that incorporating the FA score with traditional AML prognostic factors could increase the precision of survival prediction compared to using the single traditional prognostic factors alone.

### Association between the fatty acid metabolism signature and the clinical features of AML

**Fig. 5:**
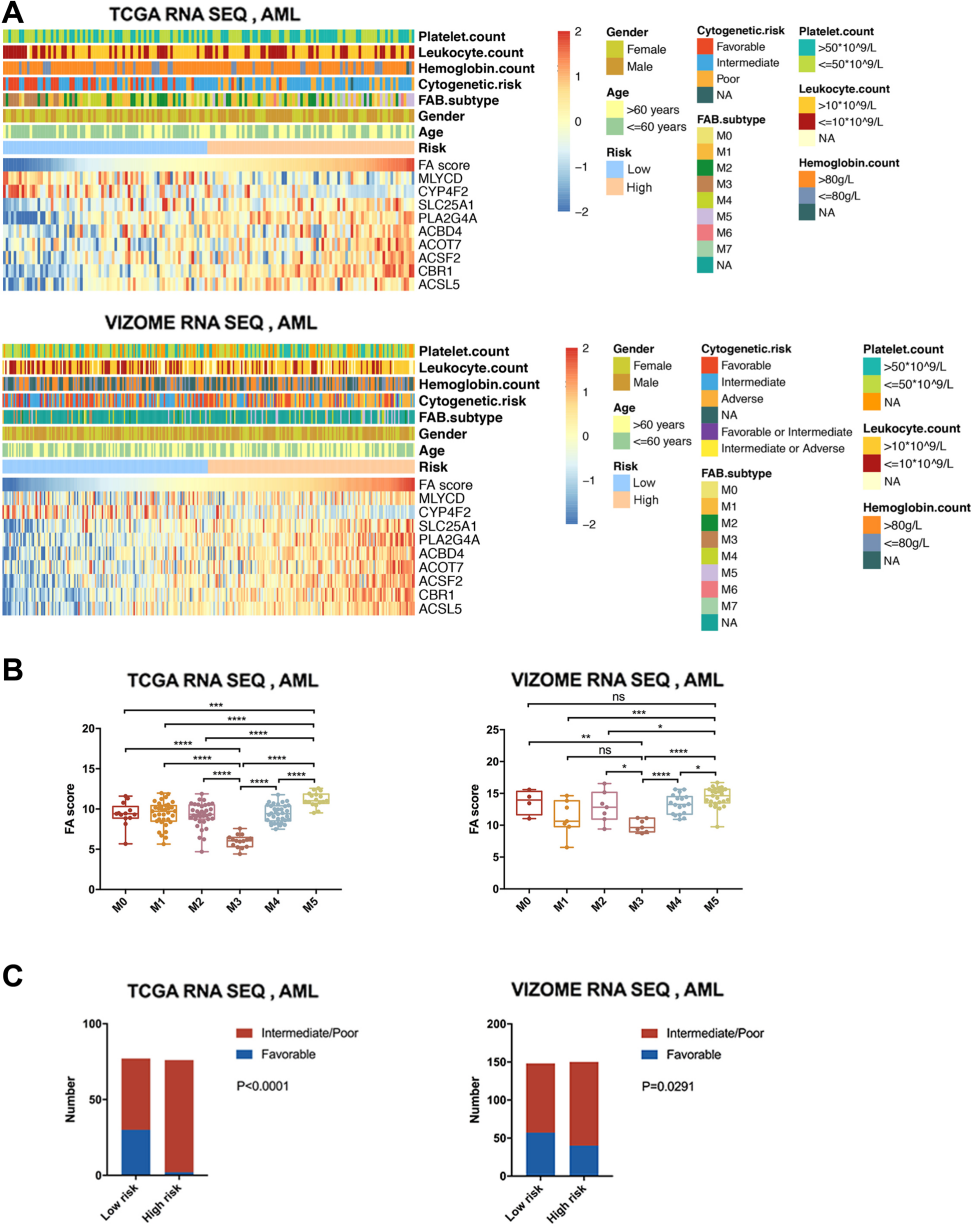
The correlation between the fatty acid metabolism signature and clinicopathological features. **A** Heatmaps described the association of the signature with age, gender, FAB subtype, cytogenetic risk, leukocyte count, hemoglobin count and platelet count in training and validation cohort. **B** The FA scores of FAB subtypes in training and validation cohort (with t test). **C** The distribution of cytogenetic risk between high-risk and low-risk group (with Chi-square test). ns, no significance; *, *P* < 0.05; **, *P* < 0.01; ***, *P* < 0.001; ****, *P* < 0.0001

To explore the clinical features associated with the FA metabolism signature, we stratified the AML patients into FA high-risk and FA low-risk groups according to their FA scores and assessed their clinical parameters. Genes that formed the fatty acid metabolism signature exhibited distinct expression patterns corresponding to the risk score (Fig. 5A). Moreover, we found that the distribution of the FAB types and cytogenetics-based risk groups were different between the FA high- and low-risk groups, while other clinical features showed no significance (Fig. 5A). Then, we analyzed the FA risk values among the FAB subtypes and found that the M5 subtype exhibited the highest risk value, while the M3 subtype (acute promylocytic leukemia) exhibited the lowest risk value (Fig. 5B). Patients with favourable cytogenetic risk were more likely classified into the FA low-risk group (Fig. 5C). We also found that patients with poor cytogenetic risk had the highest FA risk values compared with those with intermediate or favourable cytogenic risk (Supplementary Fig. 4A). These data indicated that FA risk classification were consistent with current risk factors.

### The fatty acid metabolism signature is correlated with mitochondrial metabolism, and the high-risk group exhibits an enhanced immune response

**Fig. 6:**
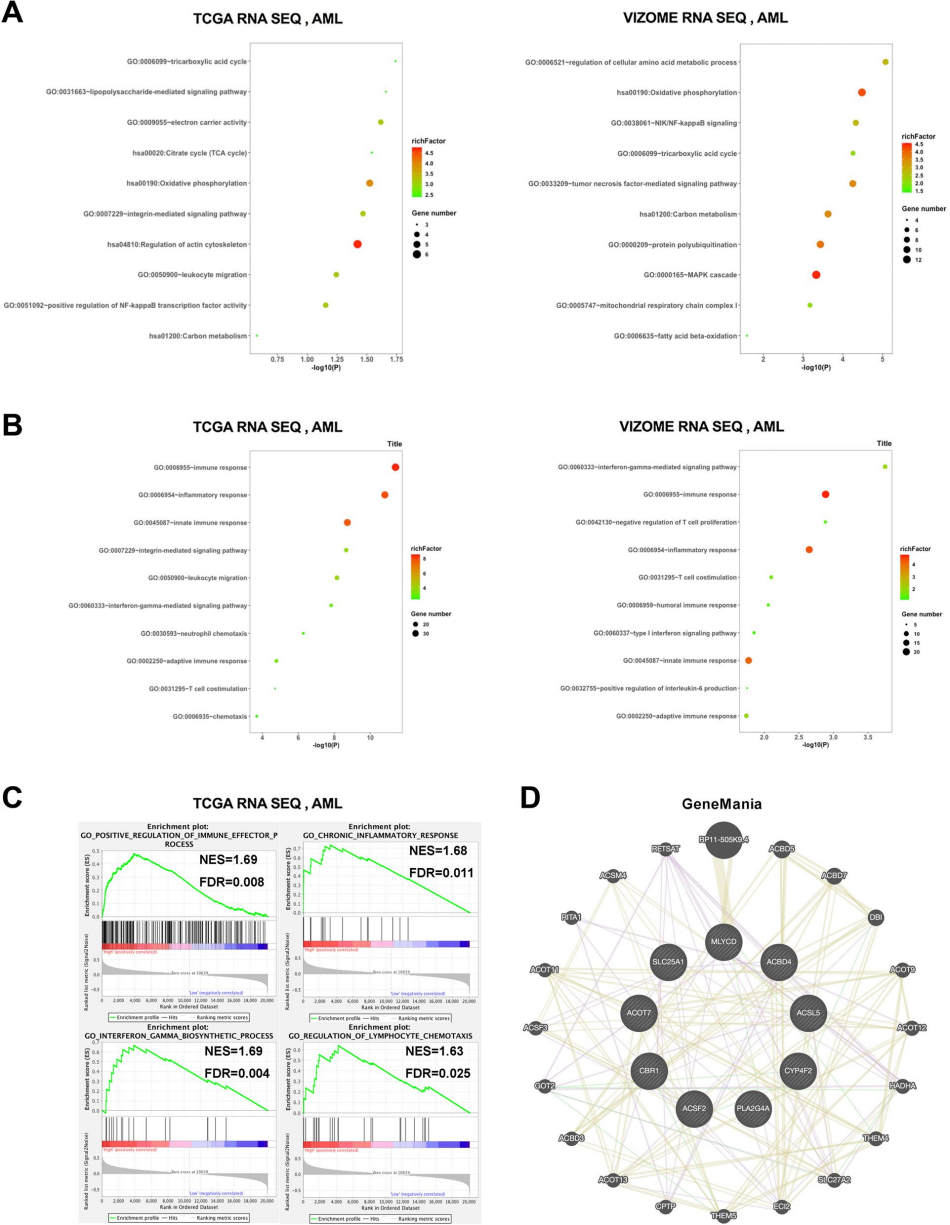
Related function analysis of the fatty acid metabolism signature. **A** GO analysis based on signature-related genes (*R* > = 0.5) showing mitochondrial metabolism associated functions of the signature in training and validation cohort. **B** GO analysis based on differential expressed genes showing inmmune associated functions of the signature in training and validation cohort. **C** The results of GSEA verified the immune-related functions of the signature in training cohort. **D** Protein-protein interaction of the nine constituent genes using GeneMANIA

To explore the related functions of the fatty acid metabolism signature, we analyzed the genes closely correlated with the FA score (*R* > = 0.5) in the TCGA and Vizome databases (Supplementary Tables 3 and 4). The results of Gene Ontology (GO) analysis showed that the signature was associated with mitochondrial metabolism, including the tricarboxylic acid (TCA) cycle and oxidative phosphorylation, in both databases (Fig. 6A). Moreover, to further investigate the differential biological functions between the high-risk and low-risk groups, we screened out differentially expressed genes (upregulated in the high-risk group; log fold change (logFC) > 0.6 in TCGA, logFC > 0.7 in Vizome; *p* < 0.05; Supplementary Tables 5 and 6). We found that most relevant biological processes were enriched in the immune response, inflammatory response and innate immune response through GO analysis (Fig. 6B). To confirm these associations, we conducted gene set enrichment analysis (GSEA) of immune-related terms, and the results showed that positive regulation of the immune effector process, IFN-γ biosynthetic process, chronic inflammatory response and regulation of lymphocyte chemotaxis were positively enriched in the high-risk group (Fig. 6C). These results suggested that the high-risk group might exhibit an enhanced immune response. In addition, we explored twenty proteins that interacted with the nine FA score proteins through GeneMANIA, and most of the twenty proteins were included in lipid metabolism pathways (Fig. 6D).

## Discussion

At present, chromosomal abnormalities and somatic gene mutations, considered the pathogenesis of AML, are combined to guide prognostic prediction and treatment selection [3, 19]. However, this evaluation system has limitations because nearly 50% of AML patients harbour a normal karyotype, and some patients even lack common somatic mutations [20]. Thus, it is essential to develop new signatures to further stratify the heterogeneous prognosis of AML patients. In this study, we constructed a suitable prognostic signature composed of genetic expression pattern involved in fatty acid metabolism in AML patients.

Previous studies have implied that fatty acid metabolism is active in LSCs and triggers various adaptive mechanisms in favour of AML cell survival [16, 21]. Reduced synthesis of monounsaturated fatty acid from saturated fatty acid leads the increased level of ROS and finally induces apoptosis of AML cells [22]. Moreover, the liver microenvironment induces fatty acid metabolism adaptation, promoting growth and chemo-resistance of liver infiltrated leukemia [23]. However, no researchers have combined the related genes of fatty acid metabolism to predict the prognosis of AML. Here, we screened the expression profile of fatty acid metabolism and identified nine genes with prognostic significance. Most of these nine genes have been reported in different tumors [24–29] and some of them have been studied in AML such as PLA2G4A, ACOT7 and CBR1 [30–32]. The detailed roles of these genes in the pathogenesis of AML require further exploration.

The fatty acid metabolism signature we established could predict the clinical outcomes of AML patients independently with preferable specificity and sensitivity.Acute monocytic leukemia (AML-M5) is a poor prognostic subtype of AML associated with hyperleukocytosis, extramedullary disease, and abnormal coagulation [33]. We found that M5 subtype patients had the highest FA scores, which suggested that fatty acid metabolism might be highly activated, providing the potential therapeutic targets. Our results showed that FA score was an independent prognostic factor and the combination of FA score, age and cytogenetic risk was superior to single factor, providing a more useful tool to stratify AML patient.

Fatty acids converge into the TCA cycle and further participate in oxidative phosphorylation (OXPHOS) in mitochondria. Several studies have suggested that the cellular enhancement of mitochondrial metabolism might induce Ara-C resistance, leading to poor prognosis and targeting OXPHOS sensitized AML cells to Ara-C [34, 35]. Thus, the desregulated fatty acid metabolism is an effective target and several inhibitors of FAO have been applied in preclinical AML studies [36]. Recently, researchers found that LSCs, which are drug-resistant cells, selectively depended on OXPHOS to supply energy and that the BCL-2 inhibitor venetoclax could inhibit OXPHOS in LSCs [37, 38]. The combination of venetoclax with the hypomethylating agent (HMA) azacitidine showed promising synergistic effects on AML patients in a phase 1b clinical study [39, 40]. Further studies showed that venetoclax combined with azacitidine targeted amino acid metabolism to inhibit OXPHOS in LSCs [41]. Moreover, up-regulation of FAO due to RAS pathway mutations or compensatory adaptation in relapsed disease attenuates the essentiality of amino acid metabolism, and finally decreases the sensitivity of the combination treatment with azacitidine and venetoclax [42]. In our study, the fatty acid metabolism signature was closely correlated with mitochondrial metabolism, which is consistent with previous studies. Based on these findings, we proposed that fatty acid inhibitors might improve the efficiency of venetoclax and azacitidine combination, especially in the patients with a high-risk FA metabolism signature.

Cellular metabolic reprogramming is not only a hallmark of tumours but also a characteristic of immune cells [43]. Long-lived memory CD8 T cells (Tm), the key factors in immunotherapy, have elevated fatty acid oxidation levels, as previous studies reported [44]. Here we found that the high-risk group showed a disturbance of immune response. Therefore, we speculated that fatty acid metabolism also played the roles in the abnormal interaction between leukemic cells and the immune cells in the bone marrow environment, resulting in immune escape and drug resistance. However, the detailed mechanism needs further exploration and validation in AML.

## Conclusion

Overall, we developed a prognostic signature based on nine fatty acid metabolism-related genes that could independently predict clinical outcomes with specificity and sensitivity, as well as improve the existing prognostic evaluation system. Moreover, the fatty acid metabolism signature might be an index to monitor the effect of targeted therapy.

## Methods

### Data collection

179 AML patients′ clinical information and transcriptome sequencing data of The Cancer Genome Atlas (TCGA) were downloaded from https://xenabrowser.net. Clinical information along with transcriptome sequencing data of VIZOME (451 patients) were downloaded from http://www.vizome.org/aml/ and http://www.cbioportal.org/. Function gene sets were obtained from http://www.gsea-msigdb.org/gsea/index.jsp.

### Bioinformatics analysis

Limma R package was used to calculate differential expression genes between high-risk and low-risk group. The gene ontology (GO) enrichment analysis was performed by DAVID 6.8 (https://david.ncifcrf.gov/tools.jsp) to find possible functions associated with the fatty acid metabolism signature. Gene set enrichment analysis (GSEA) was carried out to verify the AML-related functions between patients in high-risk and low-risk group (http://www.broadinstitute.org/gsea/index.jsp). Heatmaps were made by R language to express information correlated with the fatty acid metabolism signature. A nomogram model consists of independent prognostic factors was established for a better prediction of prognosis. The prediction accuracy of the merged system and its elements were determined by Calibration plot and C-index [45]. Protein–protein interaction among the nine genes was detected using the GeneMANIA datasets. GeneMANIA is frequently used datasets which can provide protein–protein interaction information [46].

### Statistical analysis

R language (version 3.5.2), SPSS (20.0) and GraphPad Prism 7 were mainly used for statistical analysis and figure drawing. Univariate cox regression analysis was used to identify prognostic genes. A risk signature was developed according to a linear combination of their expression levels weighted with regression coefficients from univariate cox regression analysis [47]. Kaplan-Meier survival analysis and log-rank test were used to indicate prognostic values. Multivariate cox regression analysis was carried out to identify independent prognostic factors. Chi-square test was used for showing the difference of clinical features between two groups. Two-tailed t test was performed to calculate the quantitative difference between two groups. ROC curves, forest plots and survival curves were made by GraphPad Prism 7. Statistical significance was defined as *P* value < 0.05.

## Supplementary Information

**Additional file 1: Supplementary Figure 1. **Survival curves of the nine significant genes. (A) Survival analysis revealed all the nine genes expressed prognostic value in training cohort (with log-rank test).(B) The FA score distribution in training andvalidation cohort. **Supplementary Figure 2.** The fatty acidmetabolism signature further predicted the prognosis of patients identified bytraditional prognostic markers. (A) Survival analysis revealed the signatureexpressed prognostic value in patients withage<=60 and intermediate risk in training cohort (with log-rank test). (B) Survival analysis revealed the signatureexpressed prognostic value in patients withage>60 and favorable or poor risk in training cohort (with log-rank test). (C) Survival analysisrevealed the signature expressed marginal prognostic value in patients with age<=60 and intermediate risk invalidation cohort (with log-ranktest). (D) Survival analysis revealed the signature without prognostic value in patients with age>60 and favorable or poor riskin validation cohort (with log-ranktest). **SupplementaryFigure 3.** The nomogram combined the fatty acid metabolism signature andclassic prognostic factors to predict the overall survival. (A) Nomogram plotshowed the merged score system composed of the signature, age and cytogeneticrisk in training cohort. (B) Calibration plot showed the consistency ofnomogram-predicted OS and actual OS in training cohort. (C) The C-indexcomparison between the merged score and its single composition in trainingcohort (with t test). ns, no significance; ****, *P*<0.0001.**Supplementary Figure 4.** Thecorrelation between the fatty acid metabolism signature and cytogenetic risk. (A) FA score difference among favorable,intermediate and poor risk group classified by cytogenetic risk evaluation in training and validation cohort (with t test). ns, no significance; *, *P*<0.05; ****, *P*<0.0001.  

**Additional file 2: Supplementary Table 1. **The prognostic value of fatty acid metabolism-related genes.

**Additional file 3: Supplementary Table 2.** Thirty-seven genes associated with prognosis in AML.

**Additional file 4: Supplemetary Table 3.** The genes closely correlated with the FA score (*R*>=0.5) in the TCGA database.

**Additional file 5: SupplemetaryTable 4. **The genes closely correlated with the FA score (*R*>=0.5) in the VIZOME database.

**Additional file 6: SupplementaryTable 5. **Differentially expressed genes (upregulated in the high-risk group) inthe TCGA database. 

**Additional file 7: Supplementary Table 6. **Differentially expressed genes (upregulated in the high-risk group) inthe VIZOME database.

## Data Availability

The datasets generated and/or analyzed during the current study are available in the TCGA, https://xenabrowser.net.; VIZOME, http://www.vizome.org/aml/ and http://www.cbioportal.org/; GSEA, http://www.gsea-msigdb.org/gsea/index.jsp.

## Abbreviations

AML: Acute myeloid leukemia
TCGA: The Cancer Genome Atlas
GO: Gene Ontology
GSEA: Gene set enrichment analysis
CR: Complete response
OS: Overall survival
FAO: Fatty acid oxidation
FAS: Fatty acid synthesis
LSCs: Leukemia stem cells
HRs: Hazard ratios
ROC: Receiver operating characteristic
AUCs: Areas under the curve
TCA: Tricarboxylic acid
WBC: White blood cell
OXPHOS: Oxidative phosphorylation
