# Supplementary material for: Construction and validation of a fatty acid metabolism risk signature for predicting prognosis in acute myeloid leukemia
Source: BMC Genom Data. 2022 Dec 22;23:85. doi: 10.1186/s12863-022-01099-x (PMC9784255; doi:10.1186/s12863-022-01099-x)
Supplement: Supplementary file 1 — Additional file 1: Supplementary Figure 1. Survival curves of the nine significant genes. (A) Survival analysis revealed all the nine genes expressed prognostic value in training cohort (with log-rank test).(B) The FA score distribution in training andvalidation cohort. Supplementary Figure 2. The fatty acidmetabolism signature further predicted the prognosis of patients identified bytraditional prognostic markers. (A) Survival analysis revealed the signatureexpressed prognostic value in patients withage<=60 and intermediate risk in training cohort (with log-rank test). (B) Survival analysis revealed the signatureexpressed prognostic value in patients withage>60 and favorable or poor risk in training cohort (with log-rank test). (C) Survival analysisrevealed the signature expressed marginal prognostic value in patients with age<=60 and intermediate risk invalidation cohort (with log-ranktest). (D) Survival analysis revealed the signature without prognostic value in patients with age>60 and favorable or poor riskin validation cohort (with log-ranktest). SupplementaryFigure 3. The nomogram combined the fatty acid metabolism signature andclassic prognostic factors to predict the overall survival. (A) Nomogram plotshowed the merged score system composed of the signature, age and cytogeneticrisk in training cohort. (B) Calibration plot showed the consistency ofnomogram-predicted OS and actual OS in training cohort. (C) The C-indexcomparison between the merged score and its single composition in trainingcohort (with t test). ns, no significance; ****, P<0.0001.Supplementary Figure 4. Thecorrelation between the fatty acid metabolism signature and cytogenetic risk. (A) FA score difference among favorable,intermediate and poor risk group classified by cytogenetic risk evaluation in training and validation cohort (with t test). ns, no significance; *, P<0.05; ****, P<0.0001. [file 12863_2022_1099_MOESM1_ESM.docx]

**Supplementary Figures**

**
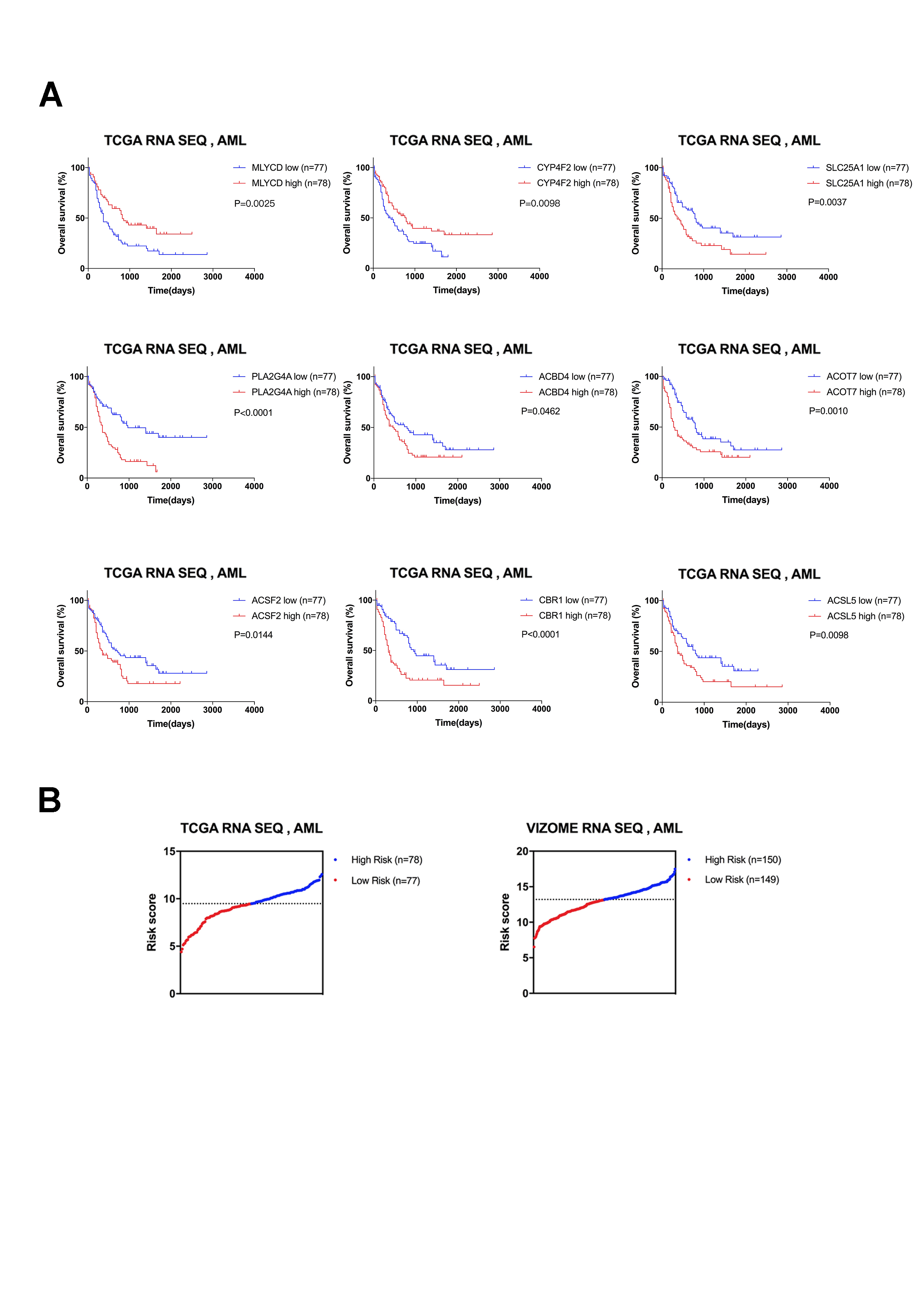
**

**Supplementary Figure 1. Survival curves of the nine significant genes.**

(**A**) Survival analysis revealed all the nine genes expressed prognostic value in training cohort (with log-rank test).

(**B**) The FA score distribution in training and validation cohort.

**
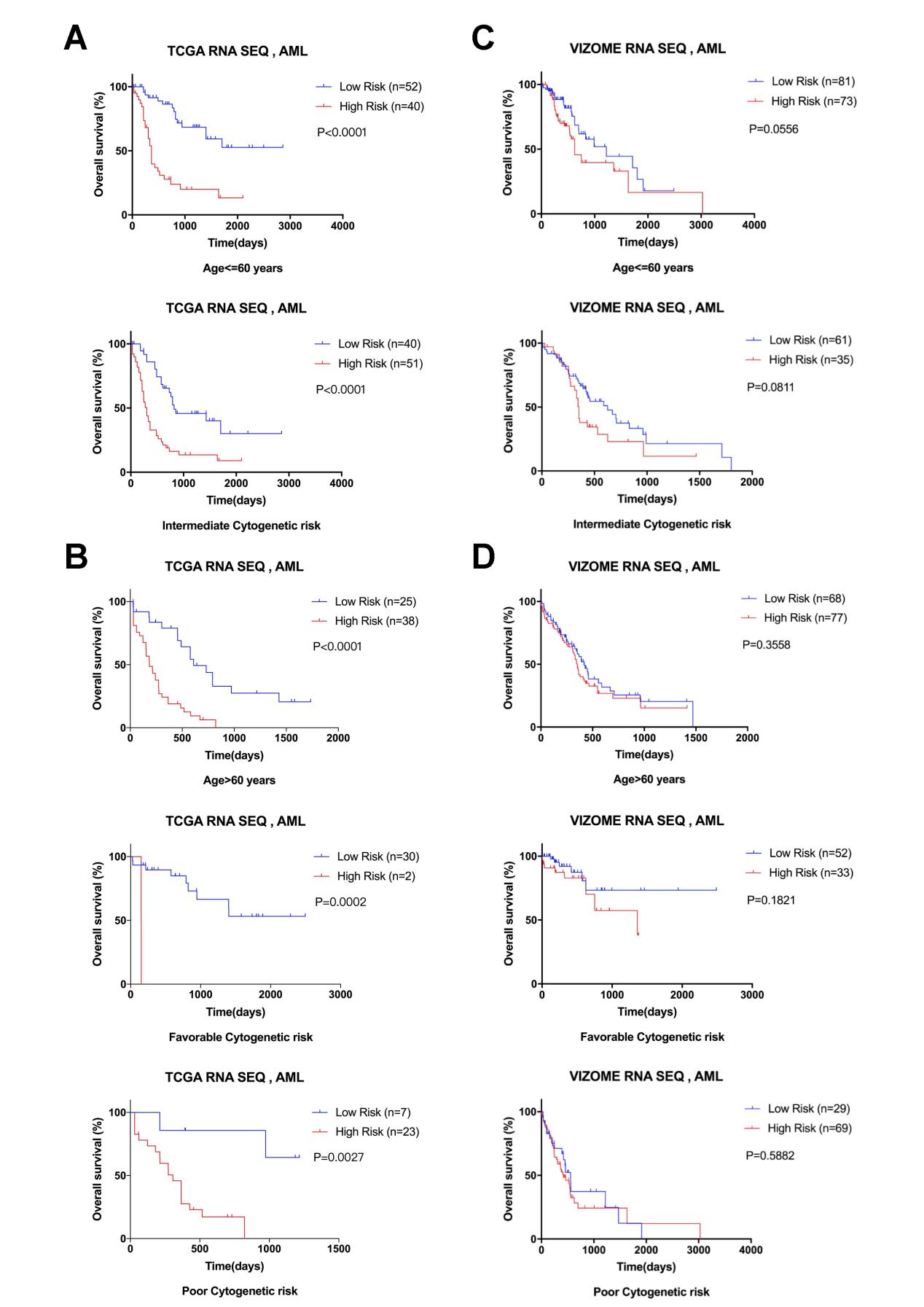
**

**Supplementary Figure 2. The fatty acid metabolism signature further predicted the prognosis of patients identified by traditional prognostic markers.**

(**A**) Survival analysis revealed the signature expressed prognostic value in patients with age<=60 and intermediate risk in training cohort (with log-rank test).

(**B**) Survival analysis revealed the signature expressed prognostic value in patients with age>60 and favorable or poor risk in training cohort (with log-rank test).

(**C**) Survival analysis revealed the signature expressed marginal prognostic value in patients with age<=60 and intermediate risk in validation cohort (with log-rank test).

(**D**) Survival analysis revealed the signature without prognostic value in patients with age>60 and favorable or poor risk in validation cohort (with log-rank test).


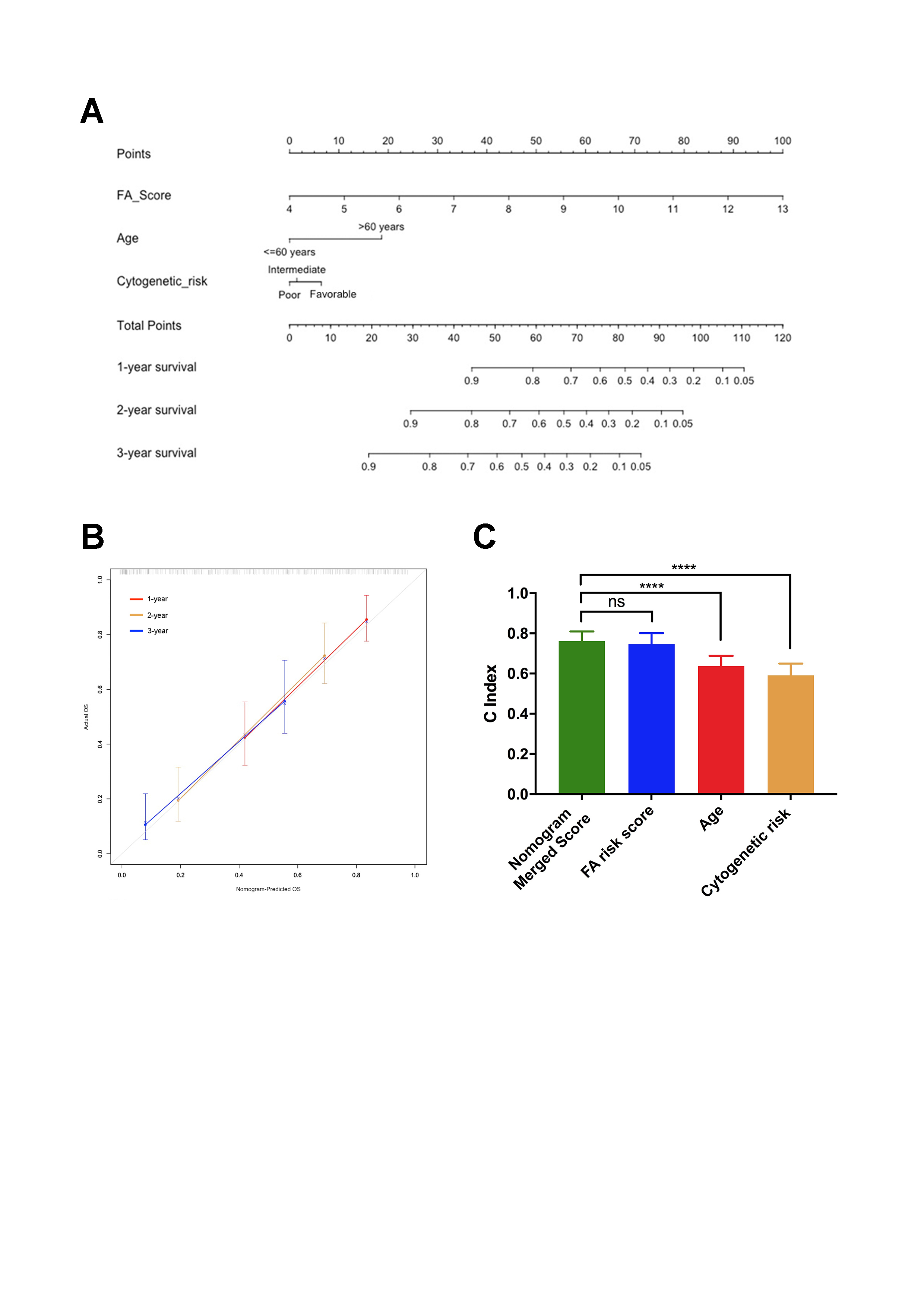


**Supplementary Figure 3. The nomogram combined the fatty acid metabolism signature and classic prognostic factors to predict the overall survival.**

(**A**) Nomogram plot showed the merged score system composed of the signature, age and cytogenetic risk in training cohort.

(**B**) Calibration plot showed the consistency of nomogram-predicted OS and actual OS in training cohort.

(**C**) The C-index comparison between the merged score and its single composition in training cohort (with t test).

ns, no significance; ****, P<0.0001


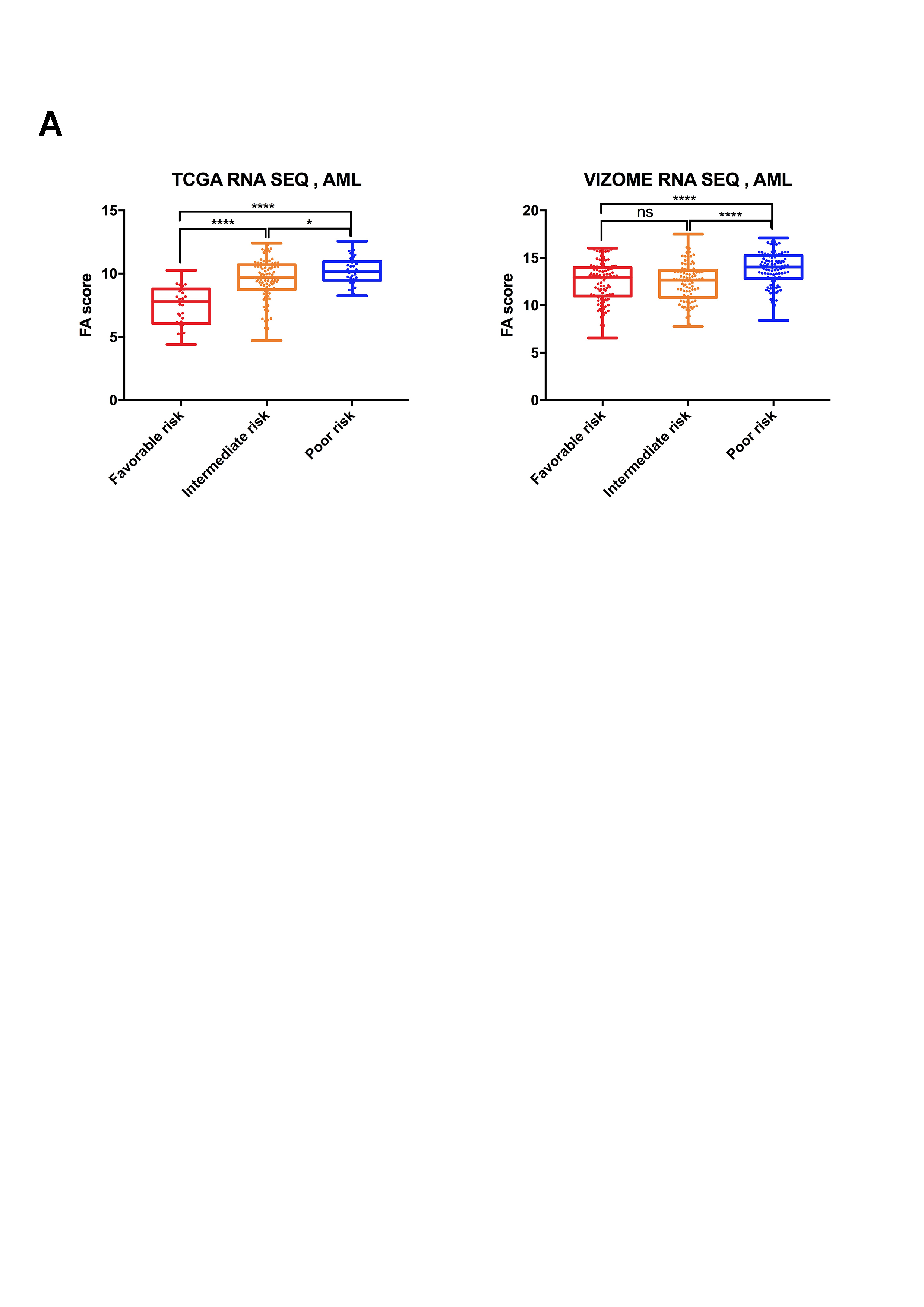


**Supplementary Figure 4. The correlation between the fatty acid metabolism signature and cytogenetic risk.**

(**A**) FA score difference among favorable, intermediate and poor risk group classified by cytogenetic risk evaluation in training and validation cohort (with t test).

ns, no significance; *, P<0.05; ****, P<0.0001.
